# Effectiveness and Minimum Effective Dose of App-Based Mobile Health Interventions for Anxiety and Depression Symptom Reduction: Systematic Review and Meta-Analysis

**DOI:** 10.2196/39454

**Published:** 2022-09-07

**Authors:** Sheng-Chieh Lu, Mindy Xu, Mei Wang, Angela Hardi, Abby L Cheng, Su-Hsin Chang, Po-Yin Yen

**Affiliations:** 1 Department of Symptom Research University of Texas MD Anderson Cancer Center Houston, TX United States; 2 Keck School of Medicine University of Southern California Los Angeles, CA United States; 3 Division of Public Health Sciences Department of Surgery Washington University in St Louis St Louis, MO United States; 4 Becker Medical Library Washington University in St Louis St Louis, MO United States; 5 Division of Physical Medicine and Rehabilitation Department of Orthopaedic Surgery Washington University in St Louis St Louis, MO United States; 6 Institute for Informatics Washington University in St Louis St Louis, MO United States; 7 Goldfarb School of Nursing Barnes Jewish College BJC HealthCare St Louis, MO United States

**Keywords:** mental health, mobile health, smartphone apps, intervention dose effectiveness, systematic review and meta-analysis

## Abstract

**Background:**

Mobile health (mHealth) apps offer new opportunities to deliver psychological treatments for mental illness in an accessible, private format. The results of several previous systematic reviews support the use of app-based mHealth interventions for anxiety and depression symptom management. However, it remains unclear how much or how long the minimum treatment “dose” is for an mHealth intervention to be effective. Just-in-time adaptive intervention (JITAI) has been introduced in the mHealth domain to facilitate behavior changes and is positioned to guide the design of mHealth interventions with enhanced adherence and effectiveness.

**Objective:**

Inspired by the JITAI framework, we conducted a systematic review and meta-analysis to evaluate the dose effectiveness of app-based mHealth interventions for anxiety and depression symptom reduction.

**Methods:**

We conducted a literature search on 7 databases (ie, Ovid MEDLINE, Embase, PsycInfo, Scopus, Cochrane Library (eg, CENTRAL), ScienceDirect, and ClinicalTrials, for publications from January 2012 to April 2020. We included randomized controlled trials (RCTs) evaluating app-based mHealth interventions for anxiety and depression. The study selection and data extraction process followed the Preferred Reporting Items for Systematic Reviews and Meta-Analyses (PRISMA) guidelines. We estimated the pooled effect size using Hedge g and appraised study quality using the revised Cochrane risk-of-bias tool for RCTs.

**Results:**

We included 15 studies involving 2627 participants for 18 app-based mHealth interventions. Participants in the intervention groups showed a significant effect on anxiety (Hedge g=–.10, 95% CI –0.14 to –0.06, I2=0%) but not on depression (Hedge g=–.08, 95% CI –0.23 to 0.07, I2=4%). Interventions of at least 7 weeks’ duration had larger effect sizes on anxiety symptom reduction.

**Conclusions:**

There is inconclusive evidence for clinical use of app-based mHealth interventions for anxiety and depression at the current stage due to the small to nonsignificant effects of the interventions and study quality concerns. The recommended dose of mHealth interventions and the sustainability of intervention effectiveness remain unclear and require further investigation.

## Introduction

More than 250 million people worldwide have depression or anxiety, which are the 2 most common mental illnesses that contribute to the global burden of disease [1]. The recent coronavirus disease pandemic has further increased the numbers of people reporting symptoms of anxiety and depression [2]. Both psychological and pharmacological therapies have been reported to effectively reduce the symptoms of mental illness. Yet, depression and anxiety disorders are notably undertreated due to a variety of barriers, such as lack of access to treatments and reluctance to get treatments because of social stigma and unawareness of symptoms [3]. The ongoing pandemic resulting in restrictions on social and physical distancing has posed additional challenges to these individuals, worsening undertreatment [2].

Mobile health (mHealth) apps leverage the ubiquity of mobile devices and the mobile-cellular telecommunication infrastructure and offer new opportunities to deliver psychological treatments for mental illness in an accessible, private format [4]. As the affordability and accessibility of smartphones are increasing, mobile apps are becoming the main component of many interventions promoting mental wellness and thus could be an exceptional tool to support mental health care delivery [5,6]. Research effort has been made to develop and examine mobile app-based interventions to improve patient engagement in symptom management and reduce mental illness symptoms. For instance, several smartphone apps are available for delivering self-directed cognitive behavioral therapy (CBT) for those with depression [7]. Other psychotherapies that are feasible to be facilitated by apps include acceptance and commitment therapy (ACT), problem-solving therapy (PST), and psychoeducation [8,9].

Several previous systematic reviews and meta-analyses have supported the use of app-based mHealth interventions for anxiety and depression symptom management. Firth et al [10,11] have reported a small-to-moderate effect size for both anxiety and depression symptom reduction following interventions delivered fully or partially by smartphone compared to control groups (anxiety: Hedge g=.33, 95% CI 0.17-0.48, *P*<.01; depression: Hedge g=.38, 95% CI 0.24-0.52, *P*<.001). Another recent systematic review reported similar results supporting the use of stand-alone smartphone apps for depression (Hedge g=.34, 95% CI 0.18-0.49, *P*<.001) and anxiety (Hedge g=.43, 95% CI 0.19-0.66, *P*≤.001) symptom reduction [12] Nevertheless, although previous studies have examined intervention features and components to identify the most effective design for app-based mHealth interventions [10,12], due to the various study lengths (ie, 4 weeks, 6 weeks, 3 months, 6 months), it remains unclear how much or how long the minimum treatment “dose” is for an mHealth intervention to be effective.

Just-in-time adaptive intervention (JITAI) has been introduced in the mHealth domain to facilitate behavior changes; it proposes the use of ongoing information (individuals’ changing status) to adapt the delivery of the intervention in its type, timing, or amount (intensity) [13]. The goal of JITAI is to increase an individual’s acceptance of the intervention as the intervention is delivered “at the moment and in the context that the person needs it most and is most likely to be receptive” [14]. Smartphones are an ideal platform to deliver JITAIs because individuals’ responses and their location can reveal whether the intervention is delivered and received at its maximum capacity. JITAI has been used to support health behaviors changes, such as physical activity [15,16], healthy diet [17,18], weight loss [19], and addiction [20-22]. A recent meta-analysis of 31 JITAI studies found significant effects of JITAI on improving health outcomes and enhancing study retention and intervention adherence [13,23,24]. JITAI emphasizes intervention tailoring to meet individual needs to achieve the best outcomes; thus, JITAI strategies regarding intervention dose (ie, type, amount, and timing of delivery) are positioned to guide the design of mHealth interventions with enhanced adherence and effectiveness.

In this study, our primary goal was to evaluate and update the evidence of app-based mHealth interventions for anxiety and depression symptom reduction through a systematic review and meta-analysis. In addition, inspired by the JITAI framework, we examined the effective mHealth dose for anxiety and depression symptoms where information was available. In other words, what is the minimum amount of usage or exposure to an mHealth app to effectively reduce anxiety and depression symptoms? To the best of our knowledge, this is the first systematic review and meta-analysis to examine the effectiveness of mHealth in anxiety and depression from a dose perspective.

## Methods

### Design

We conducted this systematic review and meta-analysis and reported the results following the Preferred Reporting Items for Systematic Reviews and Meta-Analyses (PRISMA) guidelines [25].

### Search Strategy

We searched the published literature using keywords and strategies designed by the team with the assistance of a medical librarian. These strategies were created using a combination of controlled vocabulary terms and plain keywords (Multimedia Appendix 1). Databases that were searched were Ovid MEDLINE, Embase, PsycInfo, Scopus, Cochrane Library (eg, CENTRAL), ScienceDirect, and ClinicalTrials. We limited the search to studies published from January 2012 to April 2020. All searches were completed on April 30, 2020.

### Study Selection

Studies were included if they (1) evaluated an app-based mHealth intervention designed to treat anxiety or depression or both, (2) measured symptoms of anxiety or depression, (3) were published as original research/trials in peer-reviewed journals, and (4) were written in English. We included studies that examined interventions delivered in part via mobile apps (ie, smartphone + regular phone call). We excluded studies if they (1) evaluated interventions not delivered in real-world settings (eg, only delivered within a laboratory or clinical setting), (2) evaluated interventions not delivered through a hand-held/mobile device, (3) only measured intervention usability or adherence but not the intervention effect on anxiety or depression symptoms or outcomes, (4) only measured physical stress responses but not any psychological anxiety-related symptoms, (5) did not include a control group and an experimental/comparison group with a random allocation process, or (6) used a quasi-experimental or other study design without a random allocation process.

### Quality Appraisal

We used the revised Cochrane risk-of-bias tool for randomized trials (RoB 2) [26] to assess each included study in 5 domains: (1) risk of bias arising from the randomization process, (2) risk of bias due to deviations from the intended interventions (effect of assignment to intervention), (3) risk of bias due to missing outcome data, (4) risk of bias in outcome measurement, and (5) risk of bias in selection of the reported result. The assessor rated each domain as “low risk of bias,” “some concerns,” or “high risk of bias,” which constituted an overall risk-of-bias judgment for the study. Every included study was assessed by at least 2 assessors; any discrepancies were resolved through a consensus discussion during our team meeting.

### Data Extraction

We developed a Microsoft Excel spreadsheet to facilitate systematic data extraction through iterative discussions. We extracted the following data from the included studies: study details (authors, journal, year of publication, study purposes), study design (sample size, participant eligibility criteria, control type), interventions (theoretical foundations and app components), and outcomes, including data for calculating the effect size at study endpoints and follow-ups. In addition, we obtained intervention dose design information, if available, including frequency, duration, length, and timing of delivery, to examine the minimum effective intervention dose. For outcomes, we extracted primary outcomes relevant to anxiety and depression from the included studies. If a study did not indicate the primary outcome or had multiple primary outcome measures, we used data from the most used clinically validated instruments (ie, the State-Trait Anxiety Inventory [STAI] for anxiety and the Patient Health Questionnaire-9 [PHQ-9] for depression).

### Data Synthesis

To pool the effect size of the interventions for each of the depression and anxiety measurements from the included studies with various measures, we computed Hedge g by taking the difference in the mean scores (1) between the intervention and control groups at each reported time point (between-group comparison) as well as (2) between the different time points following the interventions and the preintervention (ie, baseline) for the intervention groups (pre-post comparison). These time points included any reported time points during the interventions and during the follow-up after the conclusion of the interventions. For each comparison, we pooled and analyzed these Hedge g values for the target time point using both random-effect and fixed-effect models. The between-group and pre-post comparisons were also analyzed at the conclusion of the designed study intervention for depression and anxiety, respectively. Further, we used line graphs to visualize the pooled Hedge g values by time point, including follow-ups, to facilitate the analyses of dose-dependent effects and substantiality of the interventions.

We evaluated heterogeneity between studies using I^2^, which measures the percentage of total variance that can be explained. Study heterogeneity is considered low when I^2^<25%, moderate when I^2^ ranges from 25%-75%, and high when I^2^>75% [27]. We also visually and statistically evaluated publication bias using funnel plots and the Egger test [28]. The pooled effect accounting for missing studies was assessed using the Duval and Tweedie trim-and-fill analysis [29]. In addition, we conducted subanalyses to compare the effect sizes generated from studies that targeted both depression and anxiety symptom reduction by pooling and analyzing Hedge g values at the end of the study.

## Results

### Study Selection and Characteristics

**Figure 1:**
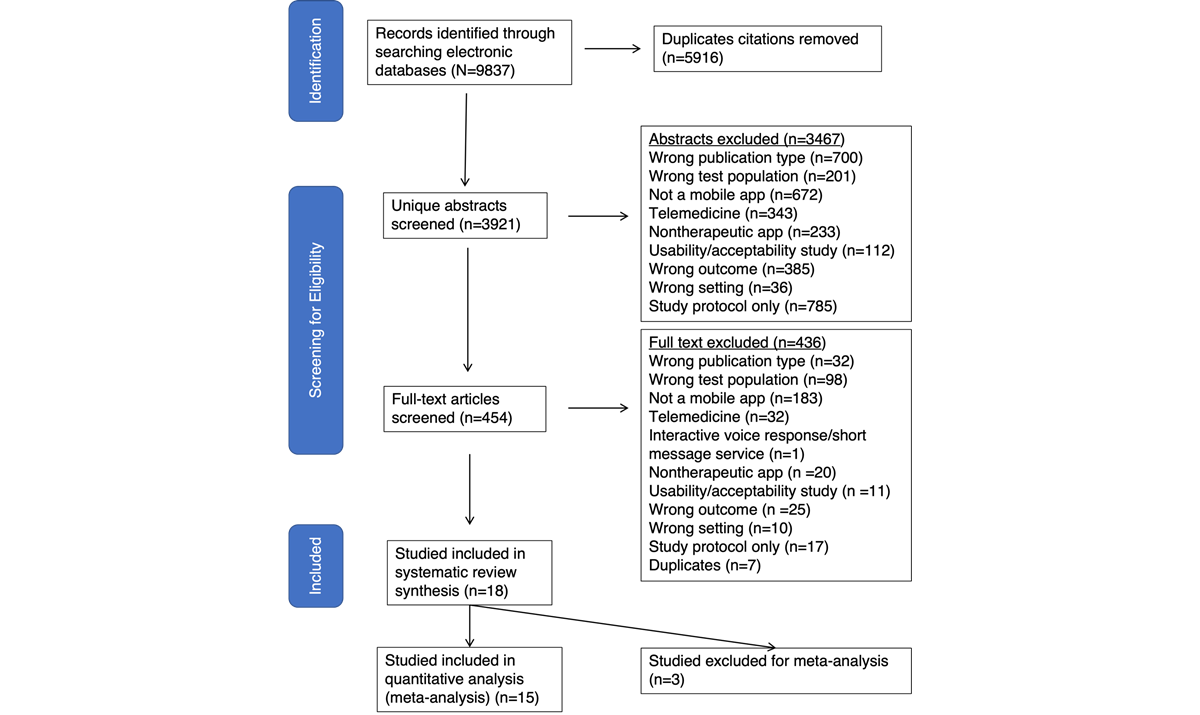
PRISMA flowchart for study selection. PRISMA: Preferred Reporting Items for Systematic Reviews and Meta-Analyses.

Our search strategy yielded a total of 9837 citations from the 7 databases, including ClinicalTrials. After removing duplicates, we screened 3921 (39.9%) abstracts and excluded 3467 (88.4%) citations that did not meet our inclusion criteria. We then reviewed 454 (11.6%) full-text articles and further excluded 436 (96%) studies based on our exclusion criteria (Figure 1). Of the remaining 18 (4%) studies, 15 (83%) were included in the meta-analysis; 3 (17%) studies did not report the data for meta-analysis [30-44].

We summarize the characteristics, interventions, and primary outcomes of the studies included in our meta-analysis (N=15) in Tables 1 and 2. A total of 1942 participants were included in the 15 studies. These studies were conducted in the United States (n=4, 26%) [32,34,38,40], Germany (n=2, 13%) [36,39], Sweden (n=2, 13%) [31,43], Australia (n=1, 7%) [35], Japan (n=1, 7%) [37], Korea (n=1, 7%) [44], Switzerland (n=1, 7%) [41], Taiwan (n=1, 7%) [42], and the United Kingdom (n=1, 7%) [33]; in addition, 1 (7%) study recruited participants worldwide (the total percentage is more than 100% due to rounding) [30].

The most frequently targeted population was adults (age≥18 years) self-reporting anxiety or depression symptoms (n=6, 40%) [30,34,36,38,40,41]. Other examined populations included university students (n=2, 13%) [33,39]. Australian indigenous youth (n=1, 7%) [35], and people with a diagnosis of cancer (n=2, 13%) [32,44], social anxiety disorder (n=1, 7%) [31], major depressive disorder (n=2, 13%) [37,43], and general anxiety disorder (GAD; n=1, 7%) [31].

A total of 18 mobile apps were examined in the studies, with 8 (44%) targeting depression symptom management, 4 (22%) targeting anxiety reduction, and 6 (34%) targeting both anxiety and depression (Table 2). The majority of the mHealth apps facilitated various CBTs (n=12, 67%) [31-33,35-39,41-44]. Other therapies included ACT (n=1, 6%) [35], mindfulness and breathing relaxation techniques (n=1, 6%) [30], self-esteem and acceptance of the present (n=1, 6%) [40], and attentional bias modification (n=1, 6%) [42]. The length of intervention ranged from 4 to 12 weeks, with 4 weeks being the most commonly used length (n=5, 28%) [30,33,36,40,42]. Most apps were designed to be used on a daily basis.

Various instruments were used as primary outcome measurements. For depression, most studies used the Beck Depression Inventory-II (BDI-II) as their primary outcome measure (n=5, 33%) [38,41-44]. Other depression assessment tools included the PHQ-9 (n=4, 27%) [34-37] and the Center for Epidemiologic Studies Depression Scale (CES-D; n=2, 13%) [39,40]. There were no common anxiety assessment tools across the studies. There were a total of 8 different measurements used in the studies, including the STAI (n=2, 13%) [42,44], the 6-item short-form of the STAI (n=2, 13%) [33,39], GAD-7 (n=1, 7%) [30], the Hamilton Anxiety Rating Scale (HAM-A; n=1, 7%) [32], the Beck Anxiety Inventory (BAI; n=1, 7%) [43], the Liebowitz Social Anxiety Scale-Self Report (LSAS-SR; n=1, 7%) [31], and the Social Interaction Anxiety Scale (SIAS; n=1, 7%) [41].

**Table 1:** Characteristics of the included studies (N=15).

Author (year), country			Study populations/eligibility criteria		Sample size			Age (years), mean (SD)		Assessment time points		Outcome measures
				Intervention, n		Control, n						
**Anxiety**												
	Pham (2016), global	Anxiety Sensitivity Index (ASI)-3≥16, Overall Anxiety Severity and Impairment Scale (OASIS)≥8, GAD-7^a^≥6		31		Waitlist: 32	18-34 (51)		Baseline, week 2, week 4 end point (EP)		GAD-7, ASI, OASIS	
	Boettcher (2018), Sweden	Diagnosis of social anxiety disorder (SAD), LSAS-SR^b^≥30		70		Bibliotherapy: 70; waitlist: 69	Intervention group (Txt): 35.4 (11.0); bibliotherapy: 35.9 (14.1); control group (Ctrl): 35.0 (11.6)		Baseline, week 3, week 7 (EP), follow-up (FU) week 3, FU week 7, FU week 9, FU week 41		LSAS-SR, PHQ^c^-9, GAD-7	
	Greer (2019), United States^d^	Age≥18 years, diagnosis of incurable solid tumor, Hospital Anxiety and Depression Scale (HADS) anxiety subscale>7, Eastern Cooperative Oncology Group (ECOG)=0-2		72		Education control: 73	Txt: 55.9 (12.4); Ctrl: 57.0 (10.1)		Baseline, week 12 (EP)		HAM-A^e^, HADS, PHQ-9	
	Ponzo (2020), United Kingdom^d^	University students, Depression Anxiety Stress Scales (DASS)-21 stress subscale>14 or DASS-21 anxiety subscale>7		72		Waitlist: 74	Txt: 19.9 (1.83); Ctrl: 19.8 (1.8)		Baseline, week 2, week 4 (EP), FU week 2		STAI^f^-S-6, PHQ-9, DASS-21	
**Depression**												
	Stile-Shields (2019), United States^d^	Age≥18 years, PHQ-9>10, Quick Inventory of Depressive Symptoms (QIDS)>11		Boost me=10; thought challenger=10		Waitlist: 10	N/R^g^		Baseline, week 3, week 6 (EP), FU week 4		PHQ-9	
	Tighe (2017), Australia	Australian indigenous youth (age 18-35 years), PHQ-9>10 or 10-item Kessler Psychological Distress Scale (K10)>25		31		Waitlist: 30	Txt: 27.5 (9.5); Ctrl: 25.0 (6.3)		Baseline, week 6 (EP)		PHQ-9	
	Ludtke (2018), Germany	Subjective need for a depression symptom reduction intervention		44		Waitlist: 44	Txt: 41.2 (11.9); Ctrl: 44.6 (10.7)		Baseline, week 4 (EP)		PHQ-9	
	Mantani (2017), Japan	Age 25-59 years, diagnosis of major depressive disorder, BDI^h^-II≥10, currently taking and resistant to 1 antidepressant		81		Medication change only: 83	Txt: 40.2 (8.8); Ctrl: 41.6 (8.9)		Baseline, week 5, week 9 (EP), FU week 8		PHQ-9, BDI-II	
	Dahne (2019), United States^d^	Age 18-65 years, PHQ-8>10		Moodivate: 24; MoodKit: 19		Treatment as usual (TAU): 9	Moodivate: 43.8 (13.3); MoodKit: 44.7 (14.0); Ctrl: 43.1 (11.9)		Week 2, week 3, week 4, week 5, week 6, week 7, week 8 (EP)		BDI-II	
**Both anxiety and depression**												
	Harrer (2018), Germany	University students, perceived stress posttreatment (PSS)-4≥8		75		Waitlist: 75	Txt: 24.0 (4.6); Ctrl: 24.2 (3.6)		Baseline, week 7 (EP), FU week 5		STAI-6, CES-D^i^	
	Roepke (2015), United States	Age≥18 years, CES-D≥16		General SB: 97; CBT^j^/positive psychotherapy SuperBetter (PPT SB): 93		Waitlist: 93	CBT/PPT SB: 42.3 (12.6); general SB: 38.0 (11.3); Ctrl: 40.3 (13.1)		Baseline, week 2, week 4 (EP), FU week 2		CES-D, GAD-7	
	Stolz (2018), Switzerland	Age≥18 years, ≥cut-off score on SIAS^k^ or Social Phobia Scale (SPS), *Diagnostic and Statistical Manual of Mental Disorders, Fourth Edition* (DSM-IV) diagnosis of SAD		60		Waitlist: 30	Txt: 34.7 (9.9); Ctrl: 35.2 (12.1)		Baseline, week 12 (EP), FU week 12		SIAS, LSAS-SR, BDI-II	
	Teng (2019), Taiwan	Age 25-35 years, PSWQ>60, DMS-IV diagnosis of GAD subscale		30		Placebo: 30; waitlist: 22	Txt: 21.5 (2.2); placebo: 21.5 (1.6); waitlist: 21.5 (1.6)		Baseline, week 2, week 3, week 4 (EP), FU week 4		STAI-S, STAI-T, BDI-II, BAI^l^	
	Ly (2015), Sweden	Age≥18 years, PHQ-9≥5, DMS-IV diagnosis of major depression		46		Face-to-face behavior activation therapy: 47	Txt: 30.2 (11.9); Ctrl: 31.0 (11.0)		Baseline, week 9 (EP), FU week 24		BDI-II, PHQ-9, BAI	
	Ham (2019), Korea^d^	Age 16-65 years, diagnosis of cancer, BDI-II≥16 or STAI>39		28		Waitlist: 26; attention control: 26	Txt: 41.9 (11.3); attention control: 43.5 (10.4); waitlist control: 47.1 (11.2)		Baseline, week 10 (EP)		BDI-II, STAI-T, STAI-S	

^a^GAD-7: Generalized Anxiety Disorder-7.

^b^LSAS-SR: Liebowitz Social Anxiety Scale-Self Report.

^c^PHQ: Patient Health Questionnaire.

^d^Studies were not included in the previous meta-analyses we identified.

^e^HAM-A: Hamilton Anxiety Rating Scale.

^f^STAI: State-Trait Anxiety Inventory.

^g^N/R: not reported.

^h^BDI: Beck Depression Inventory.

^i^CES-D: Center for Epidemiological Studies Depression Scale.

^j^CBT: cognitive behavioral therapy.

^k^SIAS: Social Interaction Anxiety Scale.

^l^BAI: Beck Anxiety Inventory.

**Table 2:** Intervention characteristics of the included studies (N=15).

Author (year), country		App contents		Intended dose		Length	Additional components
**Anxiety**							
	Pham (2016), global	Flowy app: minigames for breathing retraining with reward feedback	N/R^a^		4 weeks		N/A^b^
	Boettcher (2018), Sweden	CBT^c^ with gamification and life skill challenges	Daily use		6 weeks		Internet-based CBT with 9 modules
	Greer (2019), United States^d^	CBT with psychoeducation, activity planning, problem solving, staying present, thought creation, and summary/review	6 sessions (20-30 minutes each) with homework (10 -15 minutes each)		10-12 weeks		N/A
	Ponzo (2020), United Kingdom^d^	BioBase: CBT and self-compassion-based psychoeducational content, mood tracking, and relaxation exercises	Daily use		4 weeks		“Biobeam” wristband for passive data collection (physical activity, sleep pattern, and heart rate)
**Depression**							
	Stile-Shields (2019), United States^d^	Boost Me: behavioral activation (BA) with activity scheduling, aiming to increase rewarding activities and monitoring of mood Thought Challenger: CBT involving identifying and apprising maladaptive thoughts and creating adaptive counter thoughts	N/R		6 weeks		Weekly coaching via phone or email to enhance intervention adherence
	Tighe (2017), Australia	iBobbly: ACT^e^ with identifying thoughts, feelings, and behaviors; learning distancing techniques; regulating emotions through mindfulness, acceptance, and self-soothing activities; and identifying values, goals, personalized action plans	N/R		6 weeks		N/A
	Ludtke (2018), Germany	Good to Yourself: CBT with cognitive strategies, mindfulness, social competence skills, activating exercises	A few minutes per day		4 weeks		N/A
	Mantani (2017), Japan	Kokoro: CBT, mood monitoring, BA, and homework	1 session/week with 20 minutes/session (not including homework)		8 weeks		Antidepressant switch to escitalopram (5-10 mg/day) or to sertraline (25-100 mg/day)
	Dahne (2019), United States^d^	Moodivate: BA (psychoeducation, value identification, activity planning based on values, completion badges) MoodKit: CBT (thought identification/modification, mood tracking, journaling, activity scheduling)	At least once per day		8 weeks		N/A
**Both anxiety and depression**							
	Harrer (2018), Germany	CBT with social support, rumination, time management, procrastination, text anxiety, sleep, motivation, nutrition, exercise, mood diary, motivational messages, and online eCoach	30-90 minutes/module with 1-2 modules/week for 8 modules total		7 weeks		N/A
	Roepke (2015), United States	SuperBetter: gamified app to increase drive to accomplish goals and build social support SuperBetter“ version with CBT/positive psychotherapy (PPT): same app with additional CBT content adapted from PPT and 2 classic CBT (cognitive restructuring and behavioral activation)	10 minutes/day		4 weeks		N/A
	Stolz (2018), Switzerland	CBT with motivational enhancement, psychoeducation, cognitive restructuring, self-focused attention, behavioral experiments, summary and repetition, healthy lifestyle and problem solving, and relapse prevention	1 module/week		12 weeks		Weekly feedback from a coach
	Teng (2019), Taiwan^d^	Home-delivered attentional bias modification (HD-ABM): administers attention training for which disgusted and neutral facial expressions are used as stimuli; target “probe” replacing only the neutral face	3 times/day		4 weeks		N/A
	Ly (2015), Sweden	CBT with recall (statistics and summaries) and save important nondepressed behavior, a behavior activity database for providing suggestions, support, and inspiration; a bake-end system for therapists monitoring participants' activities; and a messaging system for communication between participants and therapists	N/R		9 weeks		Face-to-face behavior activation therapy
	Ham (2019), Korea^d^	HARUToday: CBT with psychoeducation, BA, relaxation training, cognitive restructuring, problem solving, and point reward system	10-15 minutes/session with a quiz for 48 sessions		10 weeks		N/A

^a^N/R: not reported.

^b^N/A: not applicable.

^c^CBT: cognitive behavioral therapy.

^d^Studies were not included in the previous meta-analyses we identified.

^e^ACT: acceptance and commitment therapy

### Risk-of-Bias Assessment

**Figure 2:**
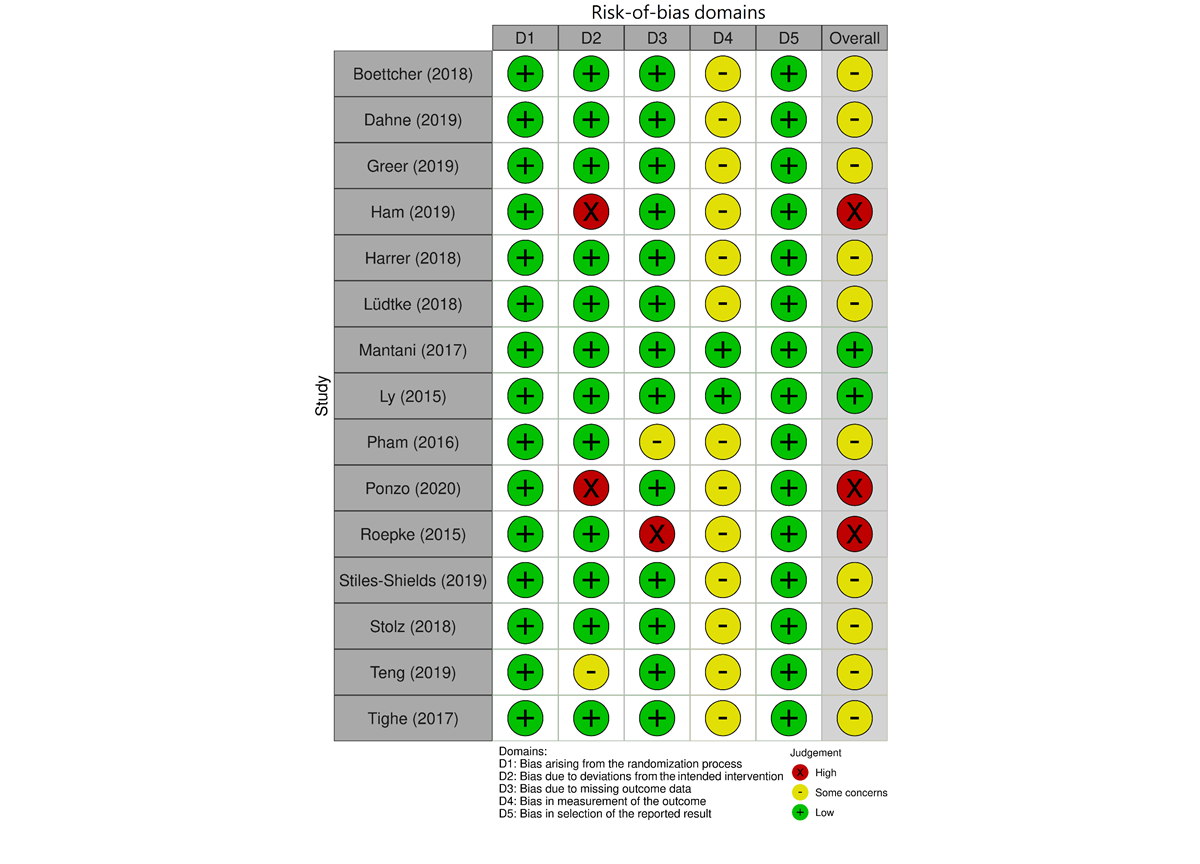
Diagram summarizing the result of our risk-of-bias evaluation among the 15 included studies using the Cochrane risk-of-bias tool for RCTs. RCT: randomized control trial.

Most studies (n=10, 67%) [30-32,34-36,38,39,41,42] were rated as “some concerns” for bias, and 3 (20%) [33,40,44] were rated as “high risk of bias” (Figure 2). All studies reported adequate randomization sequence generation and allocation concealment. In addition, 3 (20%) studies [33,42,44] reported unclear information concerning their approaches adjusting the effects of intervention nonadherence on outcomes, and 2 (13%) studies had a high attrition rate and provided no information about their approaches addressing missing data. Blinding of outcome assessment was not possible for most included studies due to the use of self-reported outcome assessments; thus, the results of most studies (n=13, 87%) [30-34,36-41,43,44], although unlikely, may be influenced by the awareness of the intervention received. Concerning outcome reporting, we found no evidence to suspect selective reporting for all studies. There was no evidence of publication bias according to the funnel plots and Egger test (Multimedia Appendix 2).

### Effectiveness of mHealth Apps in Anxiety and Depression

**Figure 3:**
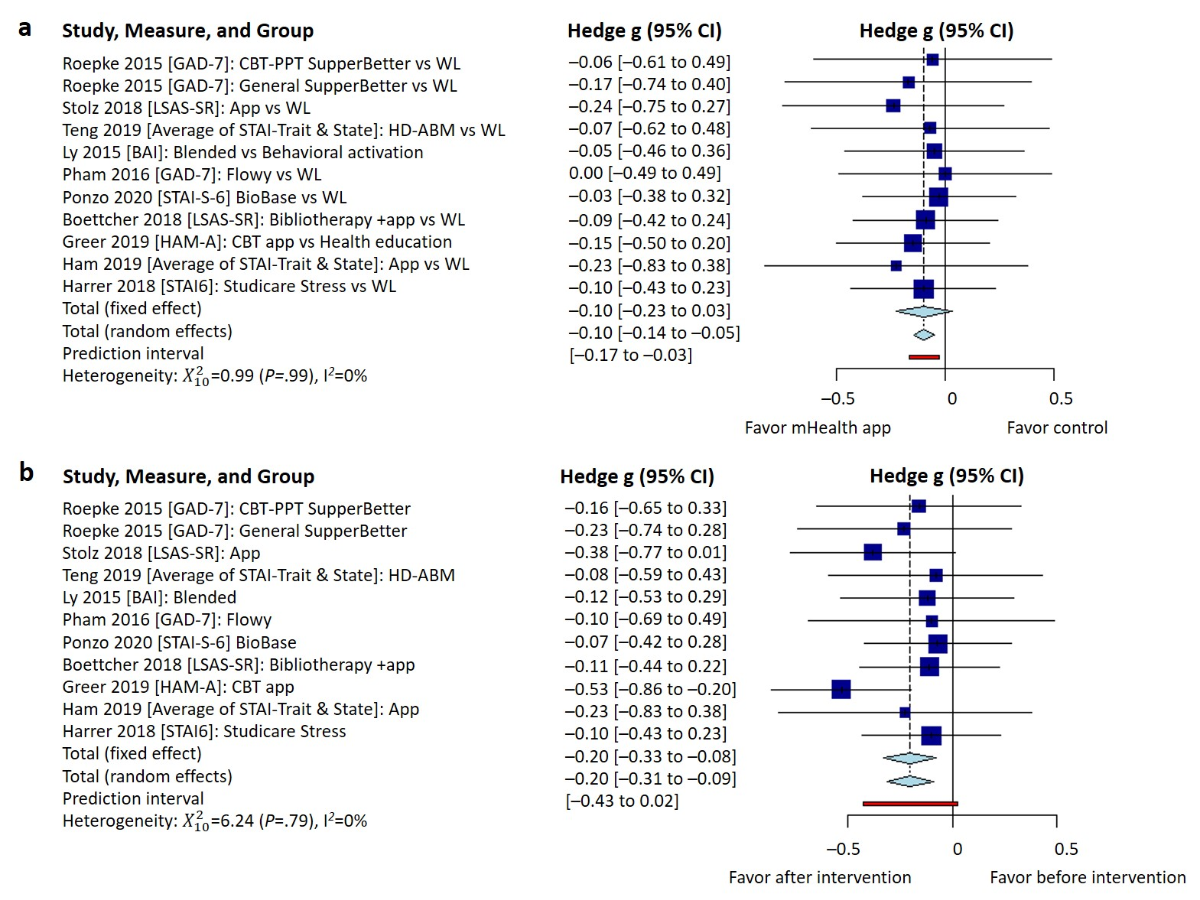
Pooled effect size of mHealth apps on anxiety symptom management at the conclusion of the intervention: (a) before-after comparison for the intervention groups and (b) comparison between intervention and control groups. BAI: Beck Anxiety Inventory; CBT: cognitive behavioral therapy; GAD: generalized anxiety disorder; HAM-A: Hamilton Anxiety Rating Scale; HD-ABM: home-delivered attentional bias modification; LSAS-SR: Liebowitz Social Anxiety Scale-Self Report; mHealth: mobile health; PPT: positive psychotherapy; STAI: State-Trait Anxiety Inventory; WL: waitlist.

Of the included 15 studies, 10 (67%) [30-33,40-42] examined the effectiveness of app-based mHealth interventions in anxiety management. When compared to the preintervention, at the conclusion of the interventions, participants receiving the interventions showed a statistically significant effect on anxiety symptoms (Hedge g=–.20, 95% CI –0.31 to –0.09, heterogeneity I^2^=0%, *P*=.79); see Figure 3a. Similarly, when compared to the control groups, at the conclusion of the interventions, participants receiving the interventions showed a statistically significant effect on anxiety symptoms (Hedge g=–.10, 95% CI –0.14 to –0.05, heterogeneity I^2^=0%, *P*>.99); see Figure 3b.

**Figure 4:**
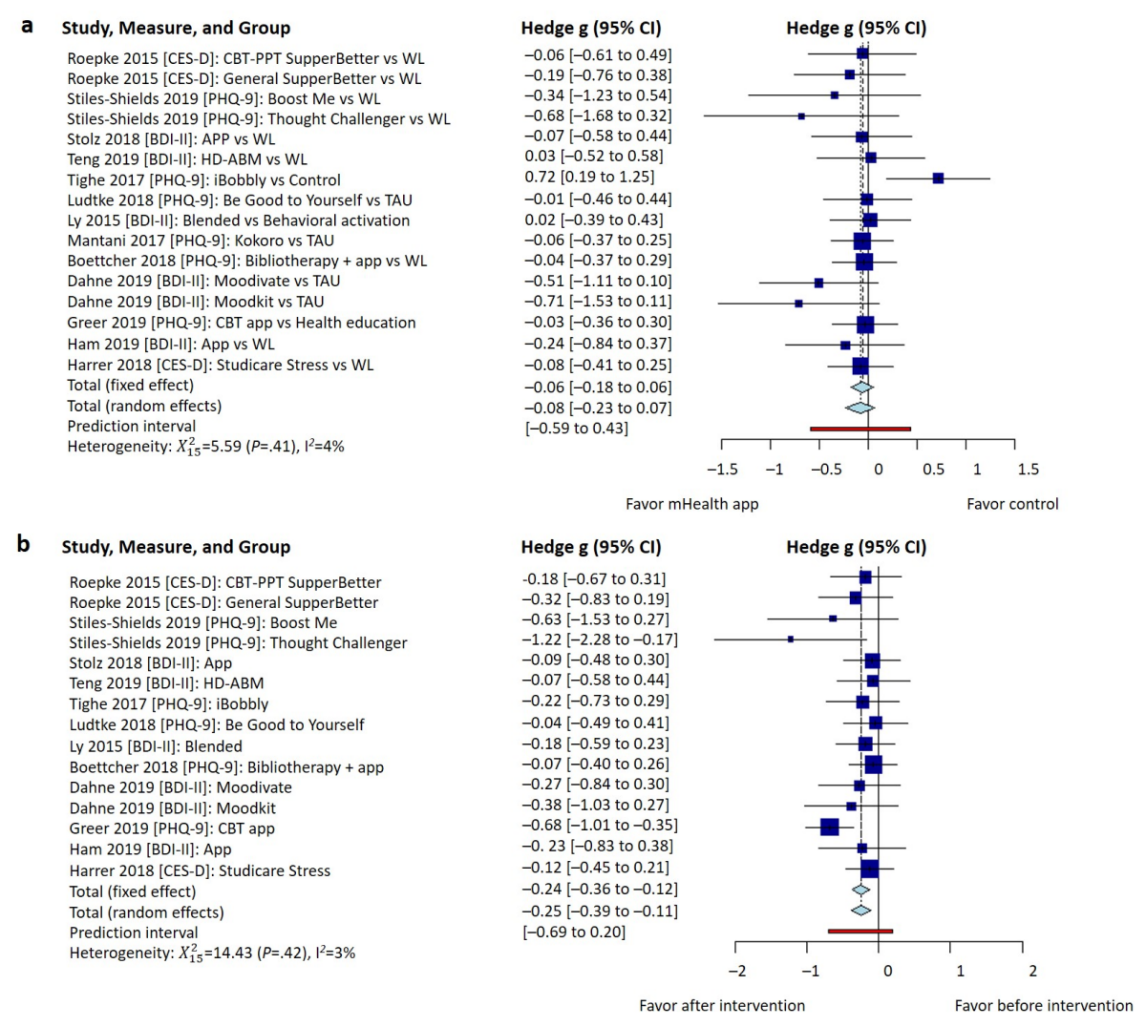
Pooled between-group effectiveness of mHealth apps on depressive symptom management: (a) before-after comparison for the intervention groups and (b) comparison between intervention and control groups. BDI: Beck Anxiety Inventory; CBT: cognitive behavioral therapy; CES-D: Center for Epidemiological Studies Depression questionnaire; HD-ABM: home-delivered attentional bias modification; mHealth: mobile health; PHQ: Patient Health Questionnaire; PPT: positive psychotherapy; TAU: treatment-as-usual; WL: waitlist.

Of the included 15 studies, 11 (73%) [32-36,39-44] evaluated the effectiveness of app-based mHealth interventions in depression management. When compared to the preintervention, at the conclusion of the interventions, participants receiving the interventions showed a statistically significant effect on depression symptoms (Hedge g=–.25, 95% CI –0.39 to –0.11, heterogeneity I^2^=3%, *P*=.42); see Figure 4a. However, when compared to the control groups, at the conclusion of the interventions, participants receiving the interventions did not show a statistically significant effect on depression symptoms (Hedge g=–.08, 95% CI –0.23 to 0.07, heterogeneity I^2^=4%, *P*=.41); see Figure 4b.

### Effects of mHealth Interventions on Depression vs Anxiety

**Figure 5:**
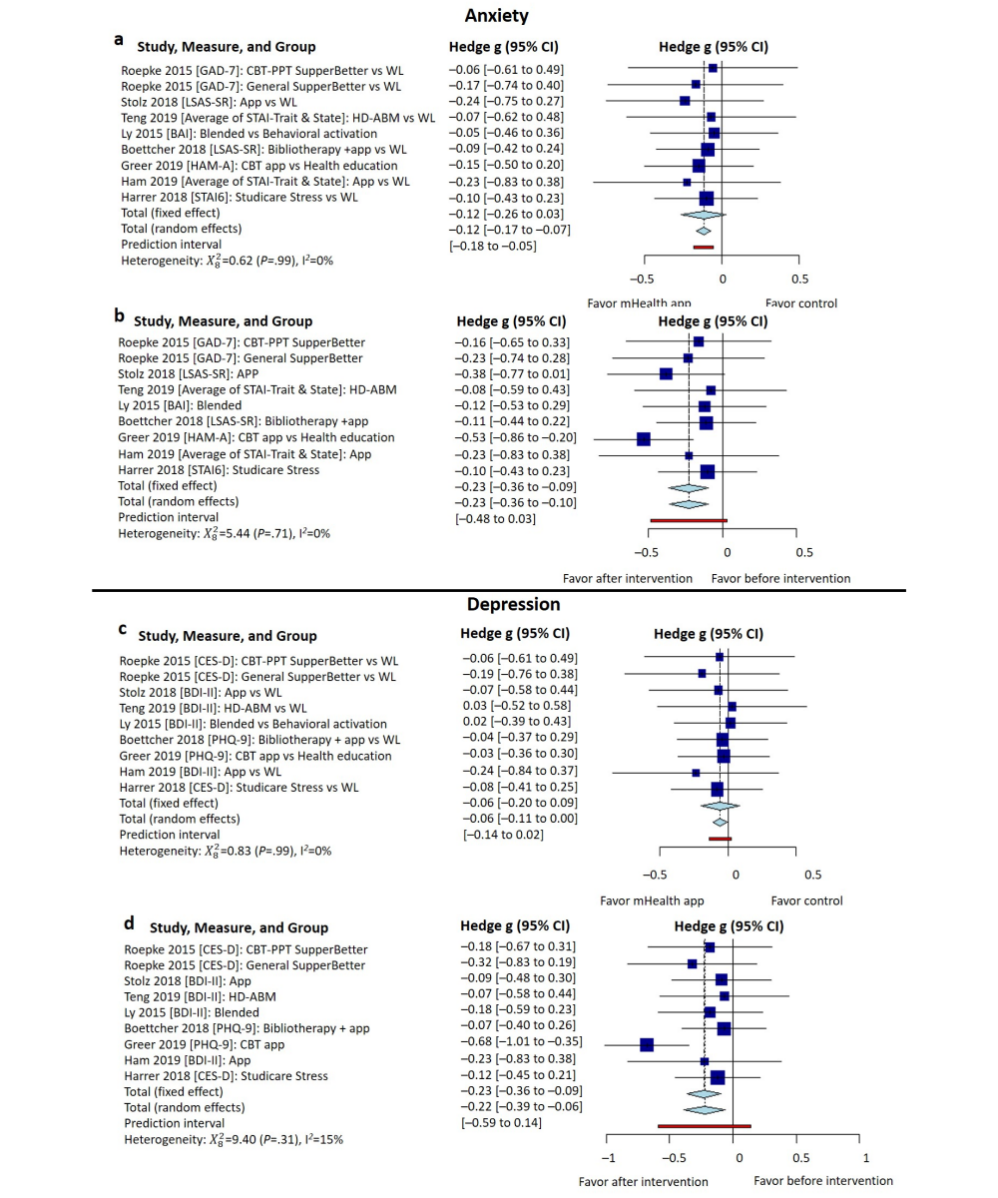
Subanalysis of pooled within-group and between-group effects of mHealth interventions on anxiety (upper panel) and depression (lower panel) from studies evaluating intervention effects on both anxiety and depression (n=8): (a) within-group comparison for the intervention groups for anxiety, (b) comparison between intervention and control groups for anxiety, (c) within-group comparison for the intervention groups for depression, and (d) comparison between intervention and control groups for depression. BAI: Beck Anxiety Inventory; BDI: Beck Anxiety Inventory; CBT: cognitive behavioral therapy; CES-D: Center for Epidemiological Studies Depression questionnaire; GAD: generalized anxiety disorder; HAM-A: Hamilton Anxiety Rating Scale; HD-ABM: home-delivered attentional bias modification; LSAS-SR: Liebowitz Social Anxiety Scale-Self Report; mHealth: mobile health; PHQ: Patient Health Questionnaire; PPT: positive psychotherapy; STAI: State-Trait Anxiety Inventory; TAU: treatment-as-usual; WL: waitlist.

Our subgroup analysis included 8 (53%) studies [31,32,39-44] evaluating the effectiveness of their interventions in both depression and anxiety. The results indicated that the intervention groups showed a significant effect on both anxiety (Hedge g=–.23, 95% CI –0.36 to –0.10, heterogeneity I^2^=0%, *P*>.99) and depression (Hedge g=–.22, 95% CI –0.39 to –0.06, heterogeneity I^2^=15%, *P*=.31) compared to baseline (Figures 5a and 5b). However, compared to the control groups (waiting list), mHealth interventions showed a significant effect only on anxiety at the conclusion of the interventions (Figure 5c) but not on depression (Figure 5d). This shows that mHealth interventions are more likely to improve anxiety but not depression.

### Dose-Dependent Effects of the mHealth Interventions

**Figure 6:**
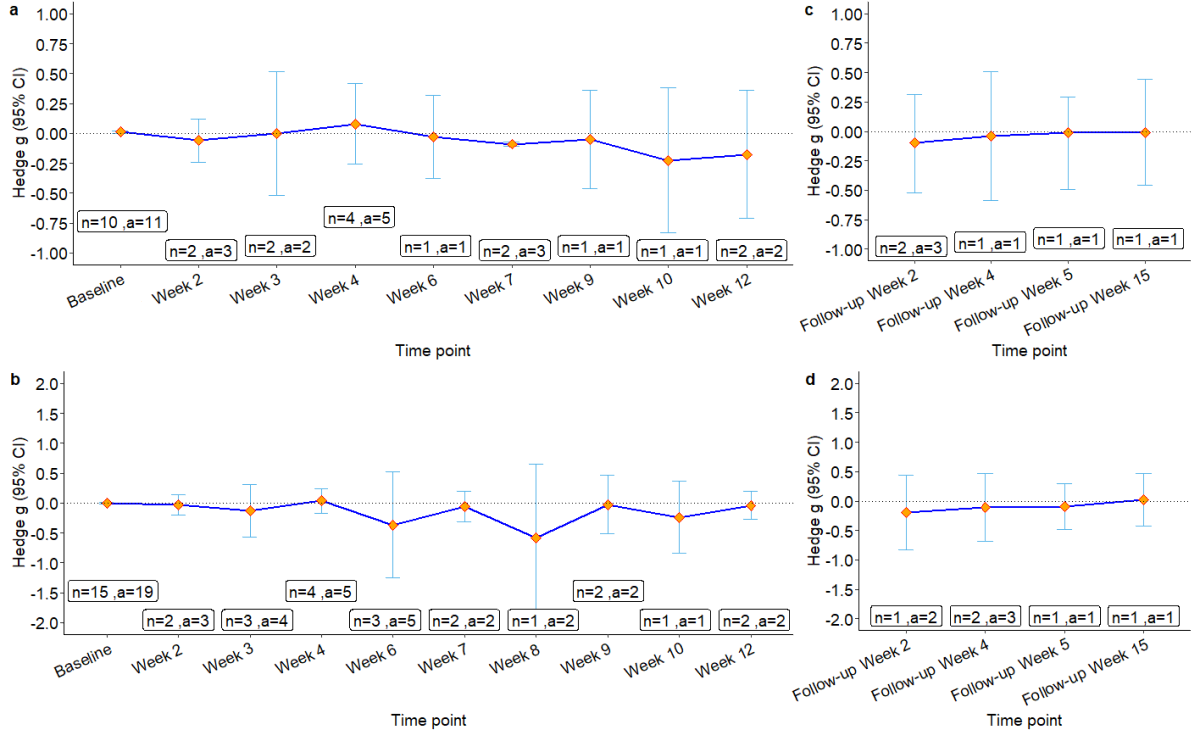
Pooled effects of the app-based mHealth intervention on anxiety (upper panel) and depression (lower panel) at different time points: (a) during the designed study intervention length and (b) during the follow-up after the designed study intervention. a: number of study arms; mHealth: mobile health.

When examining the dose-dependent effects of the mHealth interventions, interventions longer than 7 weeks had larger effect sizes on anxiety reduction, with a statistically significant effect size at week 7 (Figure 6a). In contrast, the pooled effects on depression fluctuated without a clear trend of dose-dependent effects (Figure 6b). Regarding the sustainability of intervention effects, the pooled effect sizes were not significant and reduced over time during follow-ups for both anxiety and depression (Figures 6c and 6d).

## Discussion

### Principal Findings

We conducted a systematic review and meta-analysis to examine the existing evidence on the effectiveness of app-based mHealth interventions for anxiety and depression symptom reduction. We included a total of 15 randomized controlled trials (RCTs), with many studies [32-34,38,44] published after previous reviews on a similar topic, providing an update to the current evidence. Our meta-analysis shows that app-based mHealth interventions have a modest but significant effect on anxiety reduction, consistent with previous reviews [4,11,12]. This finding adds confidence to the further development and implementation of smartphone apps to facilitate psychological treatments for anxiety symptom management [11]. In addition, our results suggest that a longer intervention (ie, 7 weeks or longer) is more likely to result in significant anxiety reduction. This finding may explain the restricted effects in studies with less than 7 weeks of app-based mHealth interventions. To the best of our knowledge, this is the first meta-analysis to assess the relationship between the app-based mHealth intervention length and the effect of the intervention. We encourage researchers to design a longer app-based mHealth intervention for anxiety symptom control and to verify our findings regarding the length of the intervention.

With regard to depression, we found that participants receiving interventions for depression experienced little symptom reduction that was not statistically significant. The finding is inconsistent with other systematic reviews reporting that smartphone apps have small-to-moderate effect sizes on depression symptom reduction [4,10,12]. The inconsistency could result from the fact that we included 1 measure per outcome per study instead of averaging the data from studies using multiple measurements for an outcome. The inconsistency may also be because previous studies included both native smartphone and web-based apps [4,10]. Web-based apps have better accessibility by allowing participants to access the interventions via various platforms [45]. In addition, the long history of web app development led to optimal user interface design, contributing to better usability and usefulness. Usability, usefulness, and accessibility have been documented as the key factors leading to successful and effective apps for mental illness management [46]. Nevertheless, we decided to exclude web-based apps because most studies reported no information about the tools their participants used to access their apps, which diminishes the purpose of our analysis on mHealth apps.

Another possible explanation of the consistency between the results of this and previous studies can be that we included 5 [32-34,38,44] studies published after previous reviews and all of them had insignificant effect sizes in our analysis. The effect sizes from the new evidence may neutralize the effect sizes from the studies included in the previous reviews. The intervention effect heterogeneity indicated that the optimal intervention content, format, and dose designs remain unclear. This is further supported by our dose-dependent analysis revealing that there is no clear relationship between the intervention length and the effect on depression, similar to a previous study [10].

There were 3 RCTs that met our eligibility criteria but were excluded from our meta-analysis due to insufficient data reported for the analysis [47-49]. All 3 studies reported positive results toward the effects of smartphone apps facilitating CBT on mental illness. Li et al [47] conducted a 12-week RCT and reported that a CBT-based smartphone chatbot intervention is efficacious for depression symptom reduction for patients with HIV and depression at both 3 and 6 months [47]. Morbeg et al [48] conducted a 4-week RCT and found that adult people receiving a CBT-based smartphone app had significantly lower anxiety and depression symptoms. Lastly, Arean et al [49] examined 2 smartphone apps in a 4-week RCT for depression and found that both apps generated a greater reduction, although not significant, in the depression symptom score compared to the control. However, we excluded these 3 studies because they either reported statistics that cannot be used to compute Hedge g without transformation based on assumptions or did not report enough data for Hedge g calculation. Studies by Li et al [47] and Morbeg et al [48] were also not included in other previous systematic reviews. The study by Arean et al [49], after data transformation with assumptions, was included in previous reviews but showed inconsistent effects on depression symptom reduction. Therefore, it was unclear whether the inclusion of these studies would alter our results for depression. Further researchers and reviewers should emphasize the gold standard of reporting to enable better study comparison and synthesis [25,50].

Consistent with previous reviews (eg, Lui et al [9]), the majority of the included studies used mobile apps to deliver CBT for anxiety or depression or both. Cognitive behavioral therapy has been delivered by computer or web apps for the treatment of various mental illnesses [51]. Our results did not suggest that smartphone apps are not useful for facilitating CBT. Rather, our results suggest that current evidence may be insufficient to guide the app-based mHealth intervention design for effective CBT-based mental illness intervention facilitation, thus requiring more research engagement. In addition, other psychotherapies, such as ACT, may also be effective in mental illness control but received relatively less attention. More studies are needed to uncover whether smartphone apps can facilitate other psychotherapies and how effective they are.

One objective of our study was to evaluate the current dose design of existing app-based mHealth interventions for anxiety and depression for an understanding of the optimal mHealth treatment length. We found that most interventions were designed to be used on a daily basis and completed within 1.5 months [52]. However, most studies provided a paucity of information about how much time their participants were asked to spend on the interventions per day or per module/session of the interventions; in addition, most studies reported no data on how much time their participants actually spent on the interventions (the actual intervention exposure). As a result, we were only able to summarize the intervention effect by the designed intervention length and dose reported in the included studies.

### Limitations and Strengths

Our review has several limitations that should be considered when interpreting the results. First, our literature search was restricted to English publications and resulted in a small amount of research available compared to other meta-analyses examining the evidence of smartphone-based interventions for mental illness. Second, the included studies used various outcome measures, and we extracted only the primary or secondary measures for anxiety and depression. Although this strategy was used in previous systematic reviews and meta-analyses on similar topics, we might have missed the effects detected by other measures. Both limitations might result in our findings of limited or nonexistent efficacy of the interventions and confidence reduction in our dose analysis results. Finally, we included 6 studies that delivered their interventions in part by smartphone. Although app components were the main parts of their interventions, our results may not represent the effects of stand-alone smartphone apps due to the inclusion of the studies. Nevertheless, we decided to include these studies because we considered these interventions were still app-based mHealth interventions. In addition, small effect sizes for 4 of the 6 studies suggest that the nonapp components do not seem to contribute to the primary effect. Further studies, including more studies for blended interventions (smartphone app + other intervention components), are needed to compare the effects of stand-alone smartphone apps and blended interventions on mental illness management.

Despite the limitations, this review has many strengths. First, our included studies covered several publications that were published after 2019 [32-34,38,44] to reflect updated evidence, which can support future development and use of app-based mHealth interventions for anxiety and depression. Second, we conducted several analyses assessing pooled intervention effects at various study time points to understand the effective length of app-based mHealth interventions. Finally, we computed the pooled effect size of the mHealth interventions during the follow-up period to uncover the sustainability of the intervention effects on anxiety and depression reduction, which was not revealed in previous systematic reviews focusing on a similar topic [10-12]. These analyses provide innovative insights informing the future study design of app-based mHealth interventions assessing for anxiety and depression symptom reduction.

### Implications for Future Studies

The dose design of app-based interventions has been suggested as an important aspect that profoundly influences intervention effects [13,24,53]. However, incomplete and inconsistent reporting of the intervention dose design and exposure in the existing studies impeded our quantitative analysis exploring the optimal intervention dose design for anxiety and depression. Future studies should explore the effect of app-based mHealth interventions with various dose designs and exposures for anxiety and depression symptom management. In addition, research efforts are needed to improve the reporting of intervention doses to enable comparable data for evidence evaluation and synthesis. The use of the JITAI framework to inform intervention design, evaluation, and reporting has potential to enable high-quality evidence for future app-based mHealth interventions for mental illness [13,24]. Finally, although most studies reported that their interventions sustained over follow-up compared to baseline, our analysis indicated that the pooled between-group effects of the interventions were not significant and rapidly reduced over time for both anxiety and depression. We recommend future studies to further explore the sustainability of symptom improvements from app-based mHealth interventions for anxiety and depression at various time points, including both during the study and after study completion (follow-up).

### Conclusion

In summary, although there is some evidence in using app-based mHealth interventions for anxiety and depression symptom reduction, clinical use cannot be recommended based on this systematic review and meta-analysis due to the small to nonexistent pooled effects found in existing studies, not to mention concerns regarding study quality/reporting of the existing studies. The effects of app-based mHealth interventions may not yet be realized, as the optimal intervention dose is still unclear. Future research should consider (1) adopting a theoretical framework, such as JITAI, to inform intervention design, evaluation, and reporting to enable high-quality evidence for app-based mHealth interventions for anxiety and depression care; (2) improving the reporting of data to enable comparable data for evidence evaluation and synthesis; and (3) exploring the sustainability of treatment benefit from the mHealth interventions.

## Appendix

Multimedia Appendix 1 Search strategy for all reference databases used.

Multimedia Appendix 2 Results of the funnel plots and Egger test.

## Abbreviations

ACT: acceptance and commitment therapy
BAI: Beck Anxiety Inventory
BDI: Beck Depression Inventory
CBT: cognitive behavioral therapy
CES-D: Center for Epidemiological Studies Depression questionnaire
GAD-7: Generalized Anxiety Disorder-7
HAM-A: Hamilton Anxiety Rating Scale
JITAI: just-in-time adaptive intervention
LSAS-SR: Liebowitz Social Anxiety Scale-Self Report
mHealth: mobile health
PHQ-9: Patient Health Questionnaire-9
PRISMA: Preferred Reporting Items for Systematic Reviews and Meta-Analyses
RCT: randomized controlled trial
SIAS: Social Interaction Anxiety Scale
STAI: State-Trait Anxiety Inventory

## References

[1] GBD 2017 Disease and Injury Incidence and Prevalence Collaborators (2018). Global, regional, and national incidence, prevalence, and years lived with disability for 354 diseases and injuries for 195 countries and territories, 1990–2017: a systematic analysis for the Global Burden of Disease Study 2017. Lancet.

[2] Mental Health America (MHA) (2021). The State of Mental Health in America.

[3] McNair BG, Highet NJ, Hickie IB, Davenport TA (2002). Exploring the perspectives of people whose lives have been affected by depression. Med J Aust.

[4] Linardon J, Cuijpers P, Carlbring P, Messer M, Fuller-Tyszkiewicz M (2019). The efficacy of app-supported smartphone interventions for mental health problems: a meta-analysis of randomized controlled trials. World Psychiatry.

[5] Torous J, Roberts LW (2017). Needed Innovation in Digital Health and Smartphone Applications for Mental Health: Transparency and Trust. JAMA Psychiatry.

[6] Miralles I, Granell C, Díaz-Sanahuja Laura, Van Woensel W, Bretón-López Juana, Mira A, Castilla D, Casteleyn S (2020). Smartphone apps for the treatment of mental disorders: systematic review. JMIR Mhealth Uhealth.

[7] Byambasuren O, Sanders S, Beller E, Glasziou P (2018). Prescribable mHealth apps identified from an overview of systematic reviews. NPJ Digit Med.

[8] Gratzer D, Strudwick G, Yeung A (2019). Mental illness: is there an app for that?. Fam Syst Health.

[9] Lui JHL, Marcus DK, Barry CT (2017). Evidence-based apps? A review of mental health mobile applications in a psychotherapy context. Professional Psychology: Research and Practice.

[10] Firth J, Torous J, Nicholas J, Carney R, Pratap A, Rosenbaum S, Sarris J (2017). The efficacy of smartphone-based mental health interventions for depressive symptoms: a meta-analysis of randomized controlled trials. World Psychiatry.

[11] Firth J, Torous J, Nicholas J, Carney R, Rosenbaum S, Sarris J (2017). Can smartphone mental health interventions reduce symptoms of anxiety? A meta-analysis of randomized controlled trials. J Affect Disord.

[12] Weisel KK, Fuhrmann LM, Berking M, Baumeister H, Cuijpers P, Ebert DD (2019). Standalone smartphone apps for mental health-a systematic review and meta-analysis. NPJ Digit Med.

[13] Nahum-Shani I, Smith SN, Spring BJ, Collins LM, Witkiewitz K, Tewari A, Murphy SA (2018). Just-in-Time Adaptive Interventions (JITAIs) in Mobile Health: Key Components and Design Principles for Ongoing Health Behavior Support. Ann Behav Med.

[14] Spruijt-Metz D, Wen CKF, O'Reilly G, Li M, Lee S, Emken BA, Mitra U, Annavaram M, Ragusa G, Narayanan S (2015). Innovations in the Use of Interactive Technology to Support Weight Management. Curr Obes Rep.

[15] King AC, Hekler EB, Grieco LA, Winter SJ, Sheats JL, Buman MP, Banerjee B, Robinson TN, Cirimele J (2013). Harnessing different motivational frames via mobile phones to promote daily physical activity and reduce sedentary behavior in aging adults. PLoS One.

[16] Hardeman W, Houghton J, Lane K, Jones A, Naughton F (2019). A systematic review of just-in-time adaptive interventions (JITAIs) to promote physical activity. Int J Behav Nutr Phys Act.

[17] Brookie KL, Mainvil LA, Carr AC, Vissers MCM, Conner TS (2017). The development and effectiveness of an ecological momentary intervention to increase daily fruit and vegetable consumption in low-consuming young adults. Appetite.

[18] Heron KE, Smyth JM (2010). Ecological momentary interventions: incorporating mobile technology into psychosocial and health behaviour treatments. Br J Health Psychol.

[19] Svetkey LP, Stevens VJ, Brantley PJ, Appel LJ, Hollis JF, Loria CM, Vollmer WM, Gullion CM, Funk K, Smith P, Samuel-Hodge C, Myers V, Lien LF, Laferriere D, Kennedy B, Jerome GJ, Heinith F, Harsha DW, Evans P, Erlinger TP, Dalcin AT, Coughlin J, Charleston J, Champagne CM, Bauck A, Ard JD, Aicher K, Weight Loss Maintenance Collaborative Research Group (2008). Comparison of strategies for sustaining weight loss: the weight loss maintenance randomized controlled trial. JAMA.

[20] Rodgers A, Corbett T, Bramley D, Riddell T, Wills M, Lin R, Jones M (2005). Do u smoke after txt? Results of a randomised trial of smoking cessation using mobile phone text messaging. Tob Control.

[21] Suffoletto B, Callaway C, Kristan J, Kraemer K, Clark DB (2012). Text-message-based drinking assessments and brief interventions for young adults discharged from the emergency department. Alcohol Clin Exp Res.

[22] Witkiewitz K, Desai SA, Bowen S, Leigh BC, Kirouac M, Larimer ME (2014). Development and evaluation of a mobile intervention for heavy drinking and smoking among college students. Psychol Addict Behav.

[23] Wang L, Miller LC (2020). Just-in-the-moment adaptive interventions (JITAI): a meta-analytical review. Health Commun.

[24] Goldstein SP, Evans BC, Flack D, Juarascio A, Manasse S, Zhang F, Forman EM (2017). Return of the JITAI: applying a just-in-time adaptive intervention framework to the development of m-Health solutions for addictive behaviors. Int J Behav Med.

[25] Page MJ, McKenzie JE, Bossuyt PM, Boutron I, Hoffmann TC, Mulrow CD, Shamseer L, Tetzlaff JM, Akl EA, Brennan SE, Chou R, Glanville J, Grimshaw JM, Hróbjartsson Asbjørn, Lalu MM, Li T, Loder EW, Mayo-Wilson E, McDonald S, McGuinness LA, Stewart LA, Thomas J, Tricco AC, Welch VA, Whiting P, Moher D (2021). The PRISMA 2020 statement: an updated guideline for reporting systematic reviews. BMJ.

[26] Sterne JAC, Savović J, Page MJ, Elbers RG, Blencowe NS, Boutron I, Cates CJ, Cheng H, Corbett MS, Eldridge SM, Emberson JR, Hernán Miguel A, Hopewell S, Hróbjartsson Asbjørn, Junqueira DR, Jüni Peter, Kirkham JJ, Lasserson T, Li T, McAleenan A, Reeves BC, Shepperd S, Shrier I, Stewart LA, Tilling K, White IR, Whiting PF, Higgins JPT (2019). RoB 2: a revised tool for assessing risk of bias in randomised trials. BMJ.

[27] Higgins JPT, Thompson SG, Deeks JJ, Altman DG (2003). Measuring inconsistency in meta-analyses. BMJ.

[28] Egger ST, Vetter S, Weniger G, Vandeleur C, Seifritz E, Müller Mario (2016). The Use of the Health of the Nation Outcome Scales for Assessing Functional Change in Treatment Outcome Monitoring of Patients with Chronic Schizophrenia. Front Public Health.

[29] Duval S, Tweedie R (2000). Trim and fill: A simple funnel-plot-based method of testing and adjusting for publication bias in meta-analysis. Biometrics.

[30] Pham Q, Khatib Y, Stansfeld S, Fox S, Green T (2016). Feasibility and Efficacy of an mHealth Game for Managing Anxiety: "Flowy" Randomized Controlled Pilot Trial and Design Evaluation. Games Health J.

[31] Boettcher J, Magnusson K, Marklund A, Berglund E, Blomdahl R, Braun U, Delin L, Lundén C, Sjöblom K, Sommer D, von Weber K, Andersson G, Carlbring P (2018). Adding a smartphone app to internet-based self-help for social anxiety: A randomized controlled trial. Computers in Human Behavior.

[32] Greer JA, Jacobs J, Pensak N, MacDonald JJ, Fuh C, Perez GK, Ward A, Tallen C, Muzikansky A, Traeger L, Penedo FJ, El-Jawahri A, Safren SA, Pirl WF, Temel JS (2019). Randomized Trial of a Tailored Cognitive-Behavioral Therapy Mobile Application for Anxiety in Patients with Incurable Cancer. Oncologist.

[33] Ponzo S, Morelli D, Kawadler JM, Hemmings NR, Bird G, Plans D (2020). Efficacy of the Digital Therapeutic Mobile App BioBase to Reduce Stress and Improve Mental Well-Being Among University Students: Randomized Controlled Trial. JMIR Mhealth Uhealth.

[34] Stiles-Shields C, Montague E, Kwasny MJ, Mohr DC (2019). Behavioral and cognitive intervention strategies delivered via coached apps for depression: Pilot trial. Psychol Serv.

[35] Tighe J, Shand F, Ridani R, Mackinnon A, De La Mata N, Christensen H (2017). Ibobbly mobile health intervention for suicide prevention in Australian Indigenous youth: a pilot randomised controlled trial. BMJ Open.

[36] Lüdtke T, Westermann S, Pult LK, Schneider BC, Pfuhl G, Moritz S (2018). Evaluation of a brief unguided psychological online intervention for depression: A controlled trial including exploratory moderator analyses. Internet Interventions.

[37] Mantani A, Kato T, Furukawa TA, Horikoshi M, Imai H, Hiroe T, Chino B, Funayama T, Yonemoto N, Zhou Q, Kawanishi N (2017). Smartphone Cognitive Behavioral Therapy as an Adjunct to Pharmacotherapy for Refractory Depression: Randomized Controlled Trial. J Med Internet Res.

[38] Dahne J, Lejuez CW, Diaz VA, Player MS, Kustanowitz J, Felton JW, Carpenter MJ (2019). Pilot Randomized Trial of a Self-Help Behavioral Activation Mobile App for Utilization in Primary Care. Behav Ther.

[39] Harrer M, Adam SH, Fleischmann RJ, Baumeister H, Auerbach R, Bruffaerts R, Cuijpers P, Kessler RC, Berking M, Lehr D, Ebert DD (2018). Effectiveness of an Internet- and App-Based Intervention for College Students With Elevated Stress: Randomized Controlled Trial. J Med Internet Res.

[40] Roepke AM, Jaffee SR, Riffle OM, McGonigal J, Broome R, Maxwell B (2015). Randomized Controlled Trial of SuperBetter, a Smartphone-Based/Internet-Based Self-Help Tool to Reduce Depressive Symptoms. Games Health J.

[41] Stolz T, Schulz A, Krieger T, Vincent A, Urech A, Moser C, Westermann S, Berger T (2018). A mobile app for social anxiety disorder: A three-arm randomized controlled trial comparing mobile and PC-based guided self-help interventions. J Consult Clin Psychol.

[42] Teng M, Hou Y, Chang S, Cheng H (2019). Home-delivered attention bias modification training via smartphone to improve attention control in sub-clinical generalized anxiety disorder: A randomized, controlled multi-session experiment. J Affect Disord.

[43] Ly KH, Topooco N, Cederlund H, Wallin A, Bergström J, Molander O, Carlbring P, Andersson G (2015). Smartphone-Supported versus Full Behavioural Activation for Depression: A Randomised Controlled Trial. PLoS One.

[44] Ham K, Chin S, Suh YJ, Rhee M, Yu E, Lee HJ, Kim J, Kim SW, Koh S, Chung K (2019). Preliminary Results From a Randomized Controlled Study for an App-Based Cognitive Behavioral Therapy Program for Depression and Anxiety in Cancer Patients. Front Psychol.

[45] United States Census Bureau (2021). Computer and Internet Use in the United States: 2018.

[46] Chan S, Torous J, Hinton L, Yellowlees P (2015). Towards a Framework for Evaluating Mobile Mental Health Apps. Telemed J E Health.

[47] Li Y, Guo Y, Hong YA, Zhu M, Zeng C, Qiao J, Xu Z, Zhang H, Zeng Y, Cai W, Li L, Liu C (2019). Mechanisms and Effects of a WeChat-Based Intervention on Suicide Among People Living With HIV and Depression: Path Model Analysis of a Randomized Controlled Trial. J Med Internet Res.

[48] Moberg C, Niles A, Beermann D (2019). Guided Self-Help Works: Randomized Waitlist Controlled Trial of Pacifica, a Mobile App Integrating Cognitive Behavioral Therapy and Mindfulness for Stress, Anxiety, and Depression. J Med Internet Res.

[49] Arean PA, Hallgren KA, Jordan JT, Gazzaley A, Atkins DC, Heagerty PJ, Anguera JA (2016). The Use and Effectiveness of Mobile Apps for Depression: Results From a Fully Remote Clinical Trial. J Med Internet Res.

[50] Agarwal S, LeFevre AE, Lee J, L'Engle K, Mehl G, Sinha C, Labrique A, WHO mHealth Technical Evidence Review Group (2016). Guidelines for reporting of health interventions using mobile phones: mobile health (mHealth) evidence reporting and assessment (mERA) checklist. BMJ.

[51] Musiat P, Tarrier N (2014). Collateral outcomes in e-mental health: a systematic review of the evidence for added benefits of computerized cognitive behavior therapy interventions for mental health. Psychol. Med.

[52] Xiong S, Berkhouse H, Schooler M, Pu W, Sun A, Gong E, Yan LL (2018). Effectiveness of mHealth Interventions in Improving Medication Adherence Among People with Hypertension: a Systematic Review. Curr Hypertens Rep.

[53] Evans W, Nielsen PE, Szekely DR, Bihm JW, Murray EA, Snider J, Abroms LC (2015). Dose-response effects of the text4baby mobile health program: randomized controlled trial. JMIR Mhealth Uhealth.

