# Supplementary material for: Effectiveness and Minimum Effective Dose of App-Based Mobile Health Interventions for Anxiety and Depression Symptom Reduction: Systematic Review and Meta-Analysis
Source: JMIR Ment Health. 2022 Sep 7;9(9):e39454. doi: 10.2196/39454 (PMC9494214; doi:10.2196/39454)
Supplement: Multimedia Appendix 1 [file mental_v9i9e39454_app1.docx]

**Appendix 1**

# The effectiveness and the minimum effective dose of application-based mobile health interventions for anxiety and depression symptom reduction: A systematic review and meta-analysis

**Systematic Review Search Methodology**

The published literature was searched using strategies designed by a medical librarian for the concepts of depression or anxiety, and mobile apps, smartphones, and other devices used for delivery of mHealth. These strategies were created using a combination of controlled vocabulary terms and plain keywords, and were executed in Ovid-Medline, Embase, PsycInfo, Scopus, Cochrane Library (including CENTRAL), ScienceDirect, and Clinicaltrials.gov. The search was limited to 2012-present (April 2020) and English. All searches were completed April 30, 2020. After duplicates were removed a total of 5600 unique citations remained for analysis. Complete search strategies are provided.

**Endnote Library Stats:**

- **Total number:** 9837
- **Number of duplicates removed:** 4441
- **Number of unique citations**: 5600 (5396 + 204 ClinicalTrials.gov results)

**Complete Search Strategies:**

**Embase.com**

=3,272 results on 4/30/2020 (limited to English and 2012-present)

('depression'/exp OR 'anxiety disorder'/exp OR depression:ti,ab OR depressive:ti,ab OR ‘dysthymic disorder’:ti,ab OR melancholia:ti,ab OR anxiety:ti,ab OR anxieties:ti,ab OR ‘acute stress disorder*’:ti,ab OR ‘distress syndrome’:ti,ab) AND ('telemedicine'/de OR 'telepsychiatry'/exp OR 'mobile health'/exp OR 'smartphone'/exp OR ‘mobile health’:ti,ab OR mHealth:ti,ab OR telehealth:ti,ab OR telepsychiatry:ti,ab OR eHealth:ti,ab OR smartphone*:ti,ab OR ‘smart phone’:ti,ab OR ‘mobile app’:ti,ab OR ‘mobile application’:ti,ab OR ‘mobile phone application’ OR ‘smartphone app’:ti,ab OR iPhone:ti,ab OR iPad:ti,ab OR ‘Android device’:ti,ab OR ‘cell phone’:ti,ab OR ‘cellular phone’:ti,ab) AND (2012:py OR 2013:py OR 2014:py OR 2015:py OR 2016:py OR 2017:py OR 2018:py OR 2019:py OR 2020:py) AND [english]/lim

**Ovid-Medline All**

= 1777 results on 4/30/2020 (Results limited to English and 2012-present)

(exp Depressive Disorder/ OR exp Anxiety/ OR depression.ti,ab. OR depressive.ti,ab. OR "dysthymic disorder".ti,ab. OR melancholia.ti,ab. OR anxiety.ti,ab. OR anxieties.ti,ab. OR "acute stress disorder*".ti,ab. OR "distress syndrome".ti,ab.) AND (Telemedicine/ OR "mobile health".ti,ab. OR mHealth.ti,ab. OR telehealth.ti,ab. OR telepsychiatry.ti,ab. OR eHealth.ti,ab. OR smartphone*.ti,ab. OR "smart phone".ti,ab. OR "mobile app".ti,ab. OR "mobile application".ti,ab. OR "mobile phone application".ti,ab. OR "smartphone app".ti,ab. OR iPhone.ti,ab. OR iPad.ti,ab. OR "Android device".ti,ab. OR "cell phone".ti,ab. OR "cellular phone".ti,ab.)

**PsycInfo (Ebsco)**

= 1,026 results on 4/30/2020 (Results limited to English, 2012-present, and Academic journals)

(((DE "Major Depression") OR DE ("Anaclitic Depression") OR (DE "Dysthymic Disorder") OR (DE "Endogenous Depression") OR (DE "Late Life Depression") OR (DE "Postpartum Depression") OR (DE "Reactive Depression") OR (DE "Recurrent Depression") OR (DE "Treatment Resistant Depression") OR (DE "Anxiety Disorders") OR (DE "Castration Anxiety") OR (DE "Death Anxiety") OR (DE "Generalized Anxiety Disorder") OR (DE "Obsessive Compulsive Disorder") OR (DE "Panic Attack") OR (DE "Panic Disorder") OR (DE "Phobias") OR (DE "Separation Anxiety Disorder") OR (DE "Trichotillomania") OR AB[depression] OR TI[depression] OR AB[depressive] OR TI[depressive] OR AB[“dysthymic disorder”] OR TI[“dysthymic disorder”] OR AB[melancholia] OR TI[melancholia] OR AB[anxiety] OR TI[anxiety] OR AB[anxieties] OR TI[anxieties] OR AB[“acute stress disorder*”] OR TI[“acute stress disorder”] OR AB[“distress syndrome”] OR TI[“distress syndrome”])) AND (((DE "Telemedicine") OR (DE "Telepsychiatry") OR (DE "Mobile Health") OR (DE "Smartphones") OR AB[“mobile health”] OR TI[“mobile health”] OR AB[mHealth] OR TI[mHealth] OR AB[telehealth] OR TI[telehealth] OR AB[telepsychiatry] OR TI[telepsychiatry] OR AB[eHealth] OR TI[ehealth] OR AB[smartphone*] OR TI[smartphone*] OR AB[“smart phone”] OR TI[“smart phone”] OR AB[“mobile app”] OR TI[“mobile app”] OR AB[“mobile application”] OR TI[“mobile application”] OR AB[“mobile phone application”] OR TI[“mobile phone application”] OR AB[“smartphone app”] OR TI[“smartphone app”] OR AB[iPhone] OR TI[iPhone] OR AB[iPad] OR TI[iPad] OR AB[“Android device”] OR TI[“android device”] OR AB[“cell phone”] OR TI[“cell phone”] OR AB[“cellular phone”] OR TI[“cellular phone”]))

**Scopus**

=2,147 results on 4/30/2020 (Results limited to English, 2012-present, and articles)

TITLE-ABS-KEY ( depression OR depressive OR {dysthymic disorder} OR melancholia OR anxiety OR anxieties OR {acute stress disorder*} OR {distress syndrome} ) AND TITLE-ABS-KEY ( {mobile health} OR mhealth OR telehealth OR telepsychiatry OR ehealth OR smartphone* OR {smart phone} OR {mobile app} OR {mobile application} OR {mobile phone application} OR {smartphone app} OR iphone OR ipad OR {Android device} OR {cell phone} OR {cellular phone} ) AND ( LIMIT-TO ( PUBYEAR , 2020 ) OR LIMIT-TO ( PUBYEAR , 2019 ) OR LIMIT-TO ( PUBYEAR , 2018 ) OR LIMIT-TO ( PUBYEAR , 2017 ) OR LIMIT-TO ( PUBYEAR , 2016 ) OR LIMIT-TO ( PUBYEAR , 2015 ) OR LIMIT-TO ( PUBYEAR , 2014 ) OR LIMIT-TO ( PUBYEAR , 2013 ) OR LIMIT-TO ( PUBYEAR , 2012 ) ) AND ( LIMIT-TO ( LANGUAGE , "English" ) ) AND ( LIMIT-TO ( DOCTYPE , "ar" ) )

**Cochrane Library**

=1368 results on 4/30/2020 (after limiting to 2012-present); All results come from CENTRAL Trials

#1 (depression OR depressive OR "dysthymic disorder" OR melancholia OR anxiety OR anxieties OR "acute stress disorder*" OR "distress syndrome"):ti,ab

#2 MeSH descriptor: [Depressive Disorder] explode all trees

#3 MeSH descriptor: [Anxiety] explode all trees

#4 #1 OR #2 OR #3

#5 MeSH descriptor: [Telemedicine] this term only

#6 ("mobile health" OR mHealth OR telehealth OR telepsychiatry OR eHealth OR smartphone* OR "smart phone" OR "mobile app" OR "mobile application" OR "mobile phone application" OR "smartphone app" OR iPhone OR iPad OR "Android device" OR "cell phone" OR "cellular phone"):ti,ab

#7 #5 OR #6

#8 #4 AND #7 with Publication Year from 2012 to 2020, with Cochrane Library publication date Between Jan 2012 and May 2020, in Trials

**Science Direct**

Note: The Science Direct platform only allows searches with 8 Boolean operators, so I had to simplify the search.

= 247 results on 4/30/2020, Limited to 2012-2020 and Review Articles and Research Articles. There doesn’t appear to be an English language limit available.

Title, abstract, keywords (depression OR anxiety) AND (mHealth OR smartphone OR "mobile app" OR “mobile application” OR iPhone OR "cell phone")

**Clinical Trials.gov**

=204 results on 4/30/2020 (limited to studies posted from 1/1/2012 to 5/1/2020)

Condition or Disease: (depression OR anxiety) AND

Intervention/Treatment: (mHealth OR smartphone OR "mobile app" OR “mobile application” OR iPhone OR "cell phone")
