# Supplementary material for: Effectiveness and Minimum Effective Dose of App-Based Mobile Health Interventions for Anxiety and Depression Symptom Reduction: Systematic Review and Meta-Analysis
Source: JMIR Ment Health. 2022 Sep 7;9(9):e39454. doi: 10.2196/39454 (PMC9494214; doi:10.2196/39454)
Supplement: Multimedia Appendix 2 [file mental_v9i9e39454_app2.docx]

**Appendix 2**

# The effectiveness and the minimum effective dose of application-based mobile health interventions for anxiety and depression symptom reduction: A systematic review and meta-analysis

**Funnel Plots and Egger’s test results**


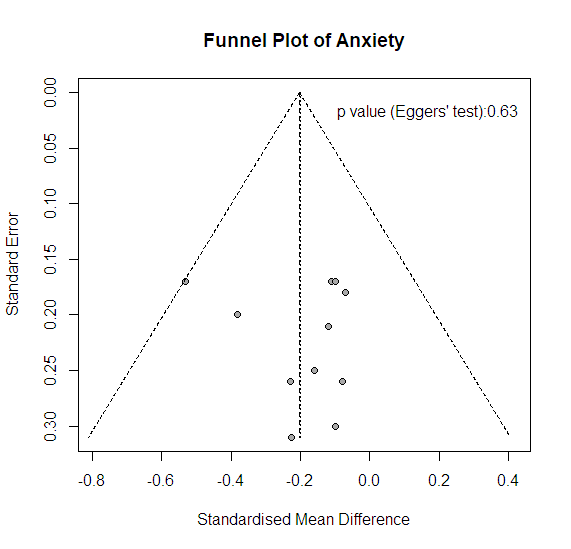


eFigure 1. Funnel plot and Egger test result of pooled effect size for anxiety symptom reduction: within-group comparison for intervention groups.


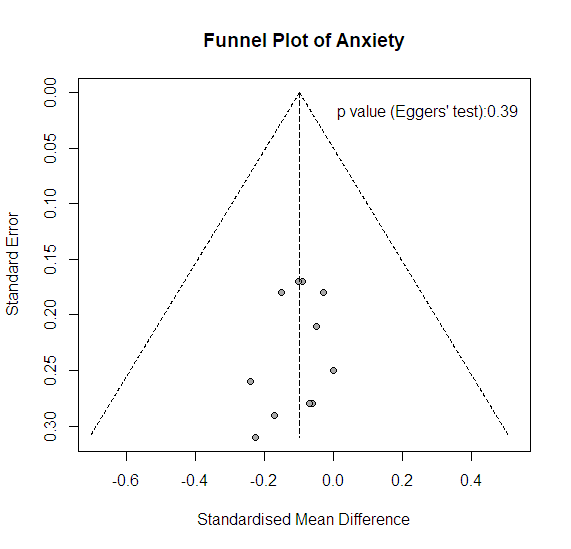


eFigure 2.Funnel plot and Egger test result of pooled effect size for anxiety symptom reduction: between-group comparison


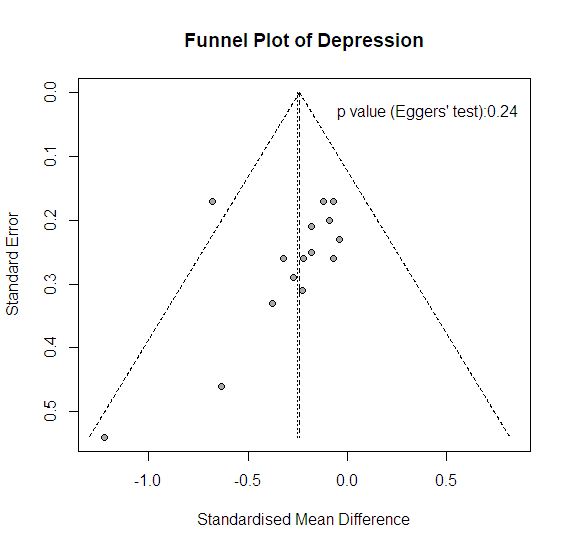


eFigure 3.Funnel plot and Egger test result for pooled effect size for depressive symptom reduction: within-group comparison for intervention groups


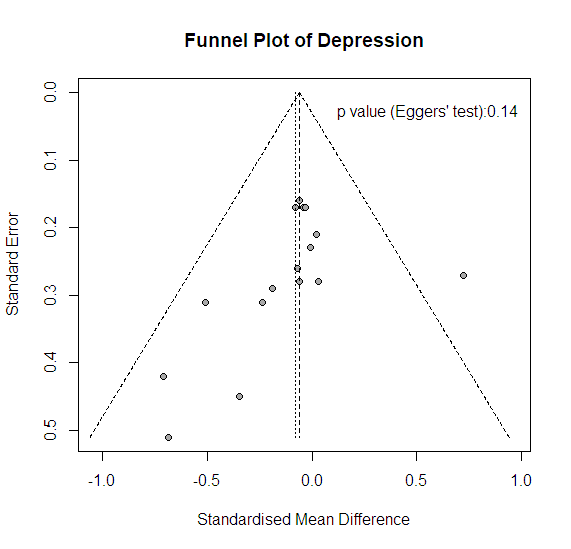


eFigure 4.Funnel plot and Egger test result for pooled effect size for depressive symptom reduction: between-group comparison for intervention groups


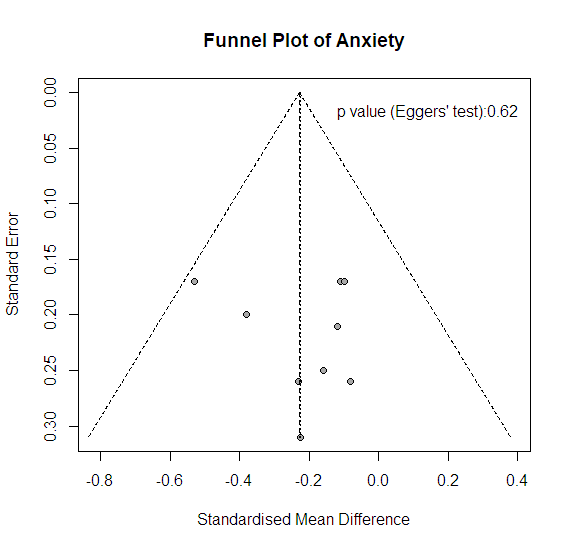


eFigure 5. Funnel plot and Egger test result for sub-analysis of pooled effect size for depressive symptom reduction: within-group comparison for the intervention groups for **anxiety.**


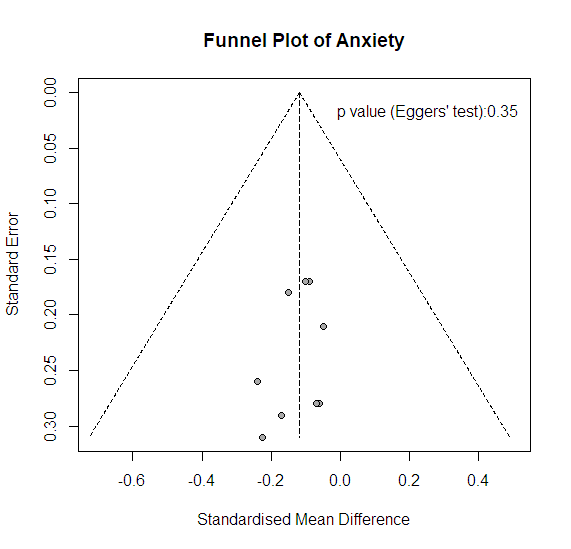


eFigure 6. Funnel plot and Egger test result for sub-analysis of pooled effect size for depressive symptom reduction: comparison between intervention and control groups for **anxiety**.


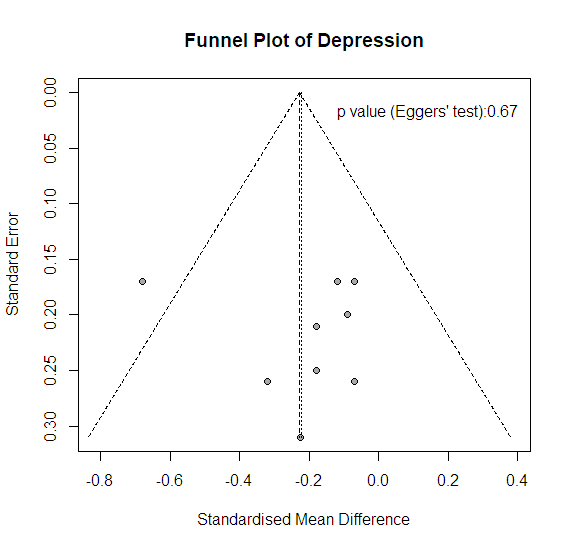


eFigure 7 Funnel plot and Egger test result for sub-analysis of pooled effect size for depressive symptom reduction: within-group comparison for the intervention groups for **depression.**


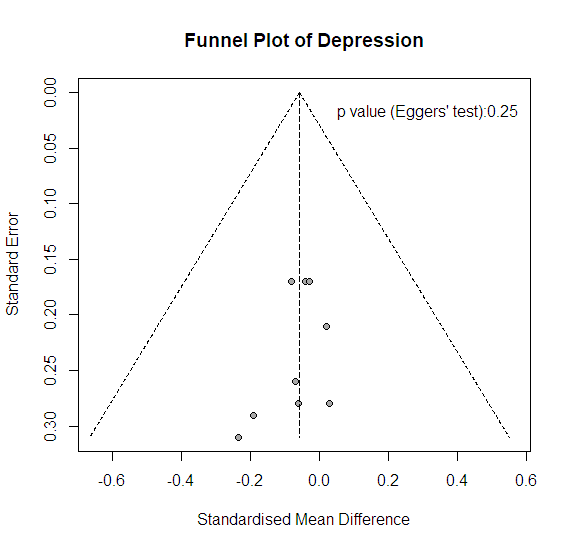


eFigure 8 Funnel plot and Egger test result for sub-analysis of pooled effect size for depressive symptom reduction: comparison between intervention and control groups for **depression**.
